# Genome-Wide Association Study of *Listeria monocytogenes* Isolates Causing Three Different Clinical Outcomes

**DOI:** 10.3390/microorganisms10101934

**Published:** 2022-09-29

**Authors:** Maria X. Cardenas-Alvarez, Daniel Restrepo-Montoya, Teresa M. Bergholz

**Affiliations:** 1 Department of Pharmacology, University of North Carolina, Chapel Hill, NC 27514, USA; 2 Max Planck Tandem Group, Universidad Nacional de Colombia, Bogotá 111321, Colombia; 3 Department of Food Science and Human Nutrition, Michigan State University, East Lansing, MI 48824, USA

**Keywords:** *Listeria monocytogenes*, listeriosis, virulence, ruminant, humans, clinical outcome, bacterial GWAS

## Abstract

Heterogeneity in virulence potential of *L. monocytogenes* subgroups have been associated with genetic elements that could provide advantages in certain environments to invade, multiply, and survive within a host. The presence of gene mutations has been found to be related to attenuated phenotypes, while the presence of groups of genes, such as pathogenicity islands (PI), has been associated with hypervirulent or stress-resistant clones. We evaluated 232 whole genome sequences from invasive listeriosis cases in human and ruminants from the US and Europe to identify genomic elements associated with strains causing three clinical outcomes: central nervous system (CNS) infections, maternal-neonatal (MN) infections, and systemic infections (SI). Phylogenetic relationships and virulence-associated genes were evaluated, and a gene-based and single nucleotide polymorphism (SNP)-based genome-wide association study (GWAS) were conducted in order to identify loci associated with the different clinical outcomes. The orthologous results indicated that genes of phage phiX174, transfer RNAs, and type I restriction-modification (RM) system genes along with SNPs in loci involved in environmental adaptation such as *rpoB* and a phosphotransferase system (PTS) were associated with one or more clinical outcomes. Detection of phenotype-specific candidate loci represents an approach that could narrow the group of genetic elements to be evaluated in future studies.

## 1. Introduction

Unique virulence features and niche specificity have been described recently for *Listeria monocytogenes* subgroups. Virulence heterogeneity among *L. monocytogenes* isolates has been reflected as a high frequency of specific clones involved in human and ruminant listeriosis in the US and Europe [1,2,3,4]. Hypervirulent and hypovirulent clones have been identified along with intrinsic characteristics that lead, for example, to better survival in the intestinal lumen [5], an increased ability to cross host barriers [6,7], or higher adaptability to food processing environments [8]. Some *L. monocytogenes* subgroups are known to cause large outbreaks, while others are the cause of sporadic cases [5,9].

This diversity in virulence within the species is mainly driven by the presence of groups of genes encoding virulence determinants, as well as polymorphisms among lineages, serogroups, and clonal complexes (CC) [10,11]. *Listeria* pathogenicity islands (LIPI) such as LIPI-3 present in certain lineage 1 strains (particularly in serotypes 1/2b and 4b), LIPI-4 that appears to be unique in CC4 strains, or the Stress Survival Islet 1 (SSI-1) that contributes to high salt and low pH tolerance, play a role in the survival and enhanced adaptation of specific *L. monocytogenes* subgroups to certain conditions [2,12,13,14,15]. Likewise, mutations in virulence or virulence-associated genes such as *InlA*, *prfA,* and *actA* contribute to attenuated phenotypes that impact the listeriosis epidemiology [3,16,17].

Given the diversity of virulence potential among *L. monocytogenes* strains, it is of interest to identify novel genetic variants that might be associated with a particular virulence phenotype, as it is likely that strains causing the same clinical manifestation share unique genetic elements associated with its ability to cause a specific clinical outcome. Although Genome-wide association studies (GWAS) have been used as a tool to associate genetic variants to specific diseases in humans, this method has recently started to be used in bacterial populations [18,19,20]. GWAS simultaneously assays genetic markers (genes, single nucleotide polymorphisms-SNPs, and *k*-mers) in the isolates and measures statistical associations between each variant and the phenotype of interest. This method has been successfully used to identify genomic features associated with host specificity in other bacterial genera such as *Campylobacter* and *Pseudomonas* [21,22], and more recently to associate *L. monocytogenes* hypervirulent and hypovirulent clones with certain ecological niches [5].

Findings in the last few years demonstrate the importance of integrating clinical, epidemiological, and experimental approaches to discover new genetic variants associated with clinically important phenotypes [2,6,23,24]. Invasive listeriosis in human and animal populations usually results in CNS infections, MN infections, or SI conditions with very high morbidity and fatality rates [25]. However, the application of GWAS for the investigation of clinically important phenotypes is still limited. Our goal was to identify genetic markers from a diverse group of listeriosis cases, that may explain the role of specific genes and SNPs in the pathogenesis processes associated with the three principal outcomes caused by *L. monocytogenes*. Knowing multiple characteristics of strain subgroups such as their clonal complexes (CC) or sequence type (ST), in addition to screening for the presence of genetic variants, could help food and health agencies to determine why certain isolates might be persisting in a specific host population or are more frequently found to be causing a particular clinical outcome.

## 2. Materials and Methods

### 2.1. Strain and Genome Collection

This study was conducted using a total of 232 *L. monocytogenes* genomes from listeriosis cases associated with three main clinical manifestations (CNS = 134, MN = 26, and SI = 72) from ruminants and humans. The dataset includes *L. monocytogenes* genomes from our in-house collection [26,27] and the Sequence Read Archive (SRA) database of The National Center for Biotechnology Information (NCBI) (Table S1). The genomes included here represent the phylogenetic diversity present in *L. monocytogenes,* as genomes from the four major lineages were included. Listeriosis cases corresponded to eight different states in the US, as well as from cases in France, Switzerland, and Great Britain. Forty-two strains were sequenced specifically for this study, while the remaining 190 were sequenced previously for other studies where the clinical outcome data was available [1,2], as well as by agencies such as the CDC and the FDA (Table S1). Six *L. monocytogenes* reference genomes belonging to lineage 1: SLCC2540 (NC_018586.1), FSL J1-220 (NC_021829.3); lineage 2: EGD-e (NC_003210.1), 10403S (NC_017544.1); lineage 3: HCC23 (NC_011660.1); and lineage 4: FSL J1-208 NZ_CM001469.1) were downloaded from GenBank (NCBI). Additionally, reference genomes from *Listeria sensu stricto* species such as *L. innocua* CLIP11262 (NC_003212.1), *L. ivanovii* PAM55 (NC_0160011.1), *L. seeligeri* SLCC3954 (NC_013891.1), and *L. welshimeri* SLCC5334 (NC_008555.1) were also downloaded from GenBank (NCBI) and included in the analyses.

### 2.2. Phenotype Designations

Listeriosis cases were grouped by clinical outcome based on the source of isolation or diagnosis reported. Terms such as rhombencephalitis, encephalitis, meningitis, brain lesion, and brain stem were grouped as central nervous system (CNS) infection. Maternal-neonatal (MN) infections grouped terms such as abortion, placenta, newborn calf, fetal, and fetus; and terms such as bacteremia, septicemia, blood, liver/lung lesion, and hip/peritoneal fluid were grouped under systemic infections (SI). Classification by clinical outcome is based on the publicly available data in the databases [1,2], as well as data reported to the CDC and FDA.

### 2.3. Whole Genome Sequencing (WGS)

Isolates were stored at −80 °C in brain-heart infusion (BHI) broth with 15% glycerol and grown in BHI broth for 20 h prior to use for DNA extraction. Genomic DNA was extracted using either the Qiagen DNeasy Blood and Tissue Kit (Qiagen, Valencia, CA, USA) or a modified phenol-chloroform protocol [28]. Quantity of the extracted DNA was assessed using the Quant-iT™ Picogreen^®^ dsDNA Assay Kit (Thermo Fisher Scientific, Carlsbad, CA, USA) and the Qbit^®^ fluorimeter (Thermo Fisher Scientific), in addition to the Nanodrop^®^ Spectrophotometer (Thermo Fisher Scientific). The Nextera^®^ XT DNA Sample Preparation Kit (Illumina, San Diego, CA, USA) was used for DNA library preparation. Paired-end whole genome sequencing (2 × 250 bp) was performed on the Illumina MiSeq system. Quality control of the reads was performed using FastQC [29] and MultiQC [30], and reads with quality values below *Phred* 20 were excluded from the analysis. De novo assembly was performed using SPAdes v. 3.10.1 (Saint Petersburg, Russia) [31] with the default settings after pre-processing the raw reads to remove low-quality bases and adapter sequences using Trimmomatic v. 0.32 (Japan) [32]. Quality of the assemblies generated by SPAdes was checked using QUAST v. 4.3 (St. Petersburg, FL, USA) [33]. Genomes that met the quality parameters recommended by Timme et al., 2020 [34] were then annotated using PROKKA v. 1.12 (Paris, France) [35] with the default parameters. A BLAST database of annotated *Listeria* strains was generated using six reference strains as described in the Prokka manual [35].

### 2.4. Lineage Determination and In Silico MLST Assignment

To classify isolates into genetic lineages, a reference tree based on core single nucleotide polymorphisms (SNPs) was generated using kSNP v. 3.1 [36]. Reference genomes for the major lineages of *L. monocytogenes* were included (SLCC2540, FSL J1-220, EGD-e, 10403S, HCC23, FSL J1-208). The resulting maximum parsimony tree (based on the consensus of 100 trees) segregated the four lineages.

To assign isolates to sequence types (ST), in silico MLST was performed using the MLST typing tool from the Center for Genomic Epidemiology (https://cge.food.dtu.dk/services/MLST/, accessed on 18 June 2019). Clonal complexes (CC) were assigned based on the Pasteur Institute Listeria database (https://bigsdb.pasteur.fr/cgi-bin/bigsdb/bigsdb.pl?db=pubmlst_listeria_isolates, accessed on 18 June 2019).

### 2.5. Pan and Core Genome Analyses and Phylogenetic Reconstruction Based on Core Genome SNPs

Annotated assemblies created by Prokka were used to calculate the core and accessory genome using Roary [37]. The core genome alignment from Roary was used as input to construct a phylogenetic tree using RaXML v. 8.2.10 [38] (run through CIPRES [39]). This phylogenetic tree was then used as the input along with the gene presence and absence matrix from the pangenome to visualize the results using Phandango v. 1.3.0 (http://jameshadfield.github.io/phandango/#/, accessed on 18 June 2019) [40].

Variant calling was performed using kSNPs v. 3.1 [36] a pipeline that aligns pair-end reads against a reference genome and identifies SNPs in the pan and core genome by a de novo assembly, estimating phylogenetic relationships based on them. Parsimony trees are created based on consensus trees from a sample of 100 trees. Kmer size used was k = 19 and it was calculated using *Kchooser,* an embedded function in kSNPs. Six reference genomes from the four major lineages were included as mentioned above. The resulting tree was re-rooted using the reference strain from lineage 4 (FSL J1-208) and edited using iTOL v. 4.4.2 [41].

### 2.6. Virulence Gene Screening

A set of sixty-one genes identified as putative or confirmed virulence factors were screened as described in previous studies (Table S2) [1,5,17]. The query gene sequences were extracted from the reference strains EGD-e (NC_003210.1 and AL591981.1), F2365 (NC_002973.6), and CLIP81459 (NC_012488.1). Genomes from reference strains from each *L. monocytogenes* lineage and from other *Listeria sensu stricto* species mentioned above were included along with the 232-listeriosis dataset. CD-HIT-STD-2D (v. 4.7) (http://weizhong-cluster.ucsd.edu/cd-hit/, accessed on 18 June 2019) was used to compare the identity between the queries and the listeriosis dataset and to calculate the sequence coverage (parameters -c.90 –n8 –S170) [42]. A gene was considered absent if: (1) the identity percentage between the query and the target sequences was <90%; (2) the gene was not found in the target sequence; (3) the gene was not complete (the maximum difference between the query and the target sequences was established as 170 nucleotides). A gene was considered present if the identity was ≥90%, and the difference between the length of the query and the target sequences was ≤170 nucleotides.

### 2.7. Gene-Based and SNP-Based GWAS

The accessory gene content of 232 *L. monocytogenes* assemblies was used to perform the gene-based GWAS using treeWAS [19], which measures the statistical association phenotype-genotype while correcting for the confounding effects of clonal population structure and homologous recombination. A file including the phenotypic variables for each individual, the gene presence/absence matrix from the pangenome calculation from Roary [37], and a phylogenetic tree that accounted for recombination calculated by RAxML v. 8.2.10 [38] (run through CIPRES [39]) and ClonalFrameML [43] were used as input data for treeWAS. Loci that were either in <10% or >90% of the strains were not included in this analysis.

The core SNPs were identified using kSNPs v. 3.1 [36] and the output was used to create a matrix of SNPs by position using an in-house script. SNPs that were either in <10% or >90% of the strains were not included in this analysis. The core SNPs matrix along with the phenotypic variables and the phylogenetic tree calculated by RAxML v. 8.2.10 [38] (run through CIPRES [39]) and ClonalFrameML [43] were used as input data for treeWAS.

## 3. Results

### 3.1. Variant Calling at the Core Genome Level and Phylogenetic Relationship Determination

**Figure 1:**
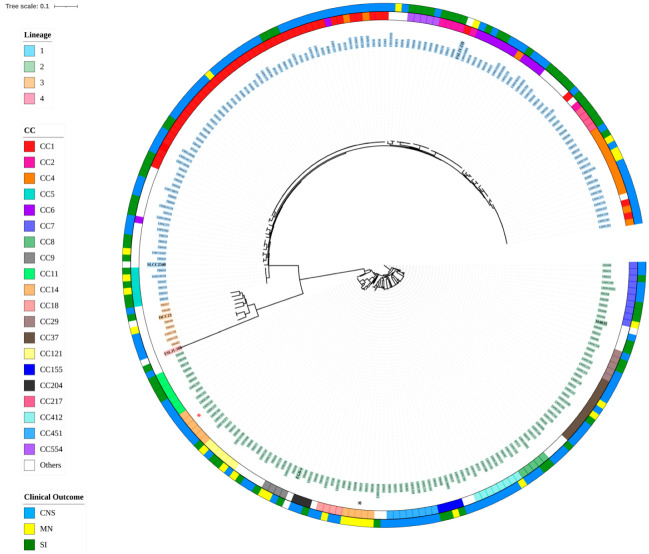
Phylogenetic tree based on core SNPs. Phylogenetic tree obtained with kSNP3 based on the core SNPs of 238 strains from lineage 1, 2, and 3, and taking FSL J1-208 from lineage 4 as an outgroup. Colors highlighting the strains’ names correspond to different lineages. CC classification is plotted in colors of the inside concentric ring, and clinical outcomes in the outside ring. Red asterisk indicates ST14/399 (CC14), while the black asterisk indicates where ST91 is located. Reference strains are bolded.

We identified a total of 18,541 core SNPs by using the completed genome of the reference strain 10403S. A tree based on the SNPs at the core genome level is shown in Figure 1. In the resulting tree, four main branches are observed, corresponding to the different lineages. CC classification showed the 20 most frequently found CCs in our dataset in different colors. Most of the strains from the same CC clustered together, however, there are individual strains that are grouped within other CCs, which is the case in some strains from CC1 (LMNC281, LMNC284, LMNC337), CC2 (TB0565), CC4 (LMNC278, LMNC331, LMNC302), and CC6 (LMNC277, LMNC357) from lineage 1 and CC14 (TB0353, TB0354, TB0407, TB0408, TB0527) from lineage 2.

### 3.2. Distribution of Virulence Genes from Listeriosis Cases Causing CNS, MN, and SI

**Figure 2:**
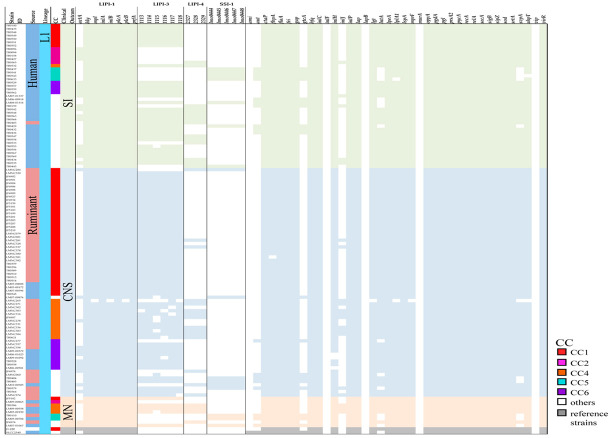
Distribution of 61 genetic elements associated with virulence and stress survival in Lineage 1. Strains are grouped by clinical outcomes (SI, CNS, MN). Colored columns on the right indicate the presence (light green, blue, and orange) or absence (white) of the genetic elements.

**Figure 3:**
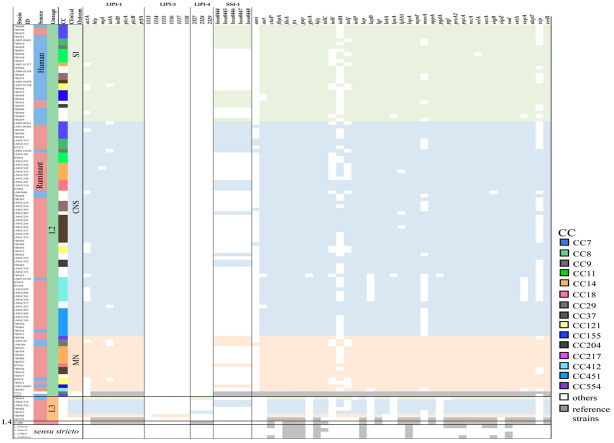
Distribution of 61 genetic elements associated with virulence and stress survival in Lineages 2, 3, and 4. Strains are grouped by clinical outcomes (SI, CNS, MN). Colored columns on the right indicate the presence (light green, blue, and orange) or absence (white) of the genetic element.

To assess the heterogeneity in virulence among clones, whole genome sequences of 242 strains (listeriosis cases: 232; reference: 10) were analyzed to evaluate the distribution of 61 genetic elements associated with virulence and stress survival (Table S2). Pathogenicity islands LIPI-1, LIPI-3, LIPI-4, and SSI-1 were included in the evaluated panel. Genes encoding LIPI-1 (*actA*, *hly*, *mpl*, *inlA*, *inlB*, *plcA*, *plcB*, and *prfA*) were present in most of the isolates as expected, however, *actA* was found partially present in 45.7% (106/232) of the isolates with gene sequences ~50% shorter than the full gene length reported. Eighty-two percent (87/106) of the absent/shorter sequences belonged to lineage 1 and from those 62.3% (66/106) were isolated from CNS infections (Figure 2 and Figure 3). Six genes from LIPI-3 (LMOf2365_1113 to _1118) were also assessed. LIPI-3 was absent in lineages 2, 3, and 4, except for one MN-associated isolate from lineage 3 that presented five out of the six LIPI-3 genes with identity percentages between 97.7% and 99.2% when compared to the reference sequences.

LIPI-4 genes were found to be present not only in CC4 isolates, but also in 14 other different CCs from lineage 1 including CC1, CC2, CC87, and CC217 showing identities above 99.85%. Furthermore, LIPI-4 was present in lineage 3 (1 human SI case and 1 ruminant CNS case) and lineage 4 (reference strain J1-208 isolated from a ruminant). SSI-1, a cluster of five genes that contributes to the survival of *L. monocytogenes* in stress conditions, was present in a total of 54 isolates (22.3%, 54/242) mostly from lineage 2 (66.7%, 36/54).

In general, genes involved in the regulation of virulence such as *prfA*, *sigB*, *virR*, *hfq*, *srtA*, and *secA* were present in all *L. monocytogenes* isolates. Unlike genes involved in vacuole lysis (i.e., *hly*, *plcA*, *plcB*, *mpl*), intracellular multiplication (i.e., *hpt*, *fri*, *relA*, *OppA*), and evasion and motility (i.e., *sod*, *inlC*, *flaA*) genes involved in adhesion (*ami*, *inlJ*, *inlF*, and *lapB*) and invasion (*aut*, *gtcA*, and *vip*) were more variable among strains. Variable genes involved in adhesion and invasion were absent, shorter, or with identity percentages below 90% in most of the lineage 1,3, and 4 isolates (except for *inlJ* also present in full length in strains from lineage 3) but present in full length in a higher proportion of lineage 2 strains (Figure 2 and Figure 3). This difference among lineages can be explained by the occurrence of alleles sharing low sequence identity as in the case of *inlF*, where the resulting protein share only 74% of the amino acid sequence identity between strains from lineage 1 and 2; or in the case of *inlJ*, where CC1 strains encode for one additional protein domain [3].

### 3.3. Pan and Core Genome Analyses

**Figure 4:**
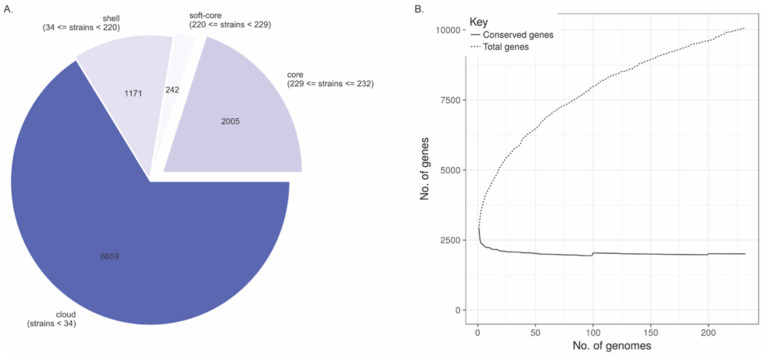
Gene cluster count among 232 *L. monocytogenes* genomes. (**A**) Classification of gene clusters among the pan genome. (**B**) Size distribution of pan genome genes related to the number of genomes included in this study.

Draft genomes of 232 *L. monocytogenes* strains were used to identify the core and accessory genes in our dataset. A total of 10,077 clusters were found: 1171 “shell” genes were present in 34–95% of the genomes; 242 “soft-core” genes were present in 95–99% of the genomes; 2005 core genes were present in 99–100% of the genomes, and 6659 genes were assigned as “cloud” genes present in less than the 15% of the genomes studied here (Figure 4A). Pan genome size distribution was also calculated according to the number of genomes analyzed: as the number of genomes included in the analysis grew, the pan genome size increased. The pan genome growth can be attributed to the increased number of accessory genes since the number of genes that belong to the core genome remains constant independently of the number of genomes included in the analysis. Although strain-specific genes are identified as accessory genes, the genomes of all *L. monocytogenes* strains seem to be similar in gene content sharing ~2000 core genes likely involved with metabolic processes, transcription, and translational processes (Figure 4B).

**Figure 5:**
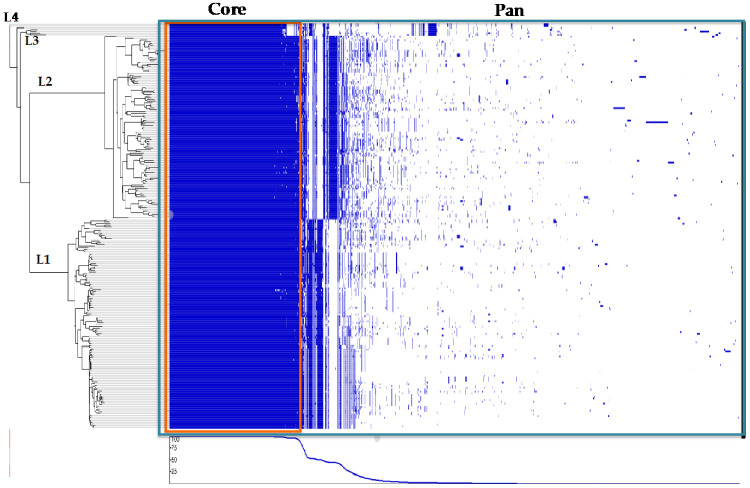
Maximum likelihood phylogenetic tree based on core genes of 232 *L. monocytogenes* isolates compared to a matrix of presence and absence of core and accessory genes. Each row represents a strain’s gene content. Each column corresponds to a gene cluster. Columns are ordered by the frequency of gene presence.

A phylogenetic tree was constructed using RaXML with the core genome alignment from Roary as the input and a gene presence and absence matrix created based on the pan genome calculated by Roary. The core genome represented around 70% (2005/2850) of the average number of genes per genome, and close to 20% (2005/10,077) of the dataset’s pan genome (Figure 5). The results indicate extensive clade-specific gene content with genes found only in subsets of strains mainly associated with lineages and CCs.

### 3.4. Gene Association with Clinical Outcomes

To identify genes associated with CNS, MN, and SI outcomes we performed a genome-wide association study (GWAS) using treeWAS [19]. About 507 genes present among the 10–90% of the isolates were analyzed. Analysis of the accessory gene presence/absence matrix resulted in the identification of a total of 14 genes associated with the three clinical outcomes evaluated. Ten genes were found to be associated with MN infections, three with SI, and one with CNS infections (this one was also found to be associated with SI) (*p* < 0.05) (Table 1, Figure S1). Table 1 also includes the total number of strains where a given gene was found (*n* = 232) independent of its association with a specific clinical outcome.

BLASTX v. 2.9.0 [44] was conducted using a non-redundant protein database. We established that among MN-associated genes, seven of them corresponded to hypothetical proteins that belong to phage phi X 174 (phiX174), a single-stranded DNA virus that infects *Escherichia coli.* Likewise, two genes corresponded to transfer RNAs (tRNA) associated with MN infections (tRNA-Phenylalanine/Alanine) and one additional associated with MN and CNS infections. These tRNAs may act as integration sites for external genetic elements. As for SI-associated genes, we identified a gene coding a hypothetical protein of unknown function and two genes related to the type I restriction-modification (RM) system (subunit M and R).

### 3.5. Core-SNPs Association with Clinical Outcomes

In *L. monocytogenes*, most virulence-associated genes identified belonged to the core genome. Therefore, we computed the core genome for our dataset and identify clinical outcome-associated SNPs. A total number of 305,337 SNPs were identified in the pan genome of our L. monocytogenes dataset. From there, 18,541 were found in the core genome and 286,796 belonged to the non-core genome. Five clinical outcome-associated SNPs were identified by treeWAS, two of them with CNS cases and tShe remaining with SI cases (*p* < 0.05) (Table 2, Figure S2).

## 4. Discussion

In bacterial populations, the main genetic variations that can influence the expression of a given phenotype are SNPs, small insertions and deletions (INDELs), gene presence-absence, copy number variations (CNVs), and sequence inversions (SIs) [45]. In response to environmental changes, bacteria not only may acquire new genetic material in protein-coding regions; they may also incorporate novel noncoding regions to enable more appropriate regulation of their existing gene repertoire [46]. Recent studies have demonstrated that SNPs within noncoding regions, such as promoters, contribute to increased virulence and survival [47,48]. In our study, we collected the genomes of 232 clinical isolates from three main listeriosis outcomes (CNS, MN, and SI) in humans and ruminants from the US and Europe to identify genes and core SNPs that vary among closely related strains and that may be potentially associated with *L. monocytogenes* clinical presentations. We recognize that not evaluating SNPs within noncoding regions is a limitation of our study, however, in the majority of bacterial genomes, between 6–14% belong to non-coding regions (12% in *E. coli*). Thus, even when SNPs within noncoding regions might be determinants of critical functions, the content of such regions is low and relatively uniform as their evolution appears to be mostly due to selective pressure to minimize the amount of non-functional DNA [49].

First, we used core genome SNPs to generate a tree to establish the phylogenetic relationships between the isolates included in this study. Four main branches corresponding to four lineages were observed and isolates were grouped in 56 CCs. As expected, strains from the same CC clustered together, with some exceptions, such as some strains from CC1, CC2, CC4, and CC6 from lineage 1 and CC14 from lineage 2. Interestingly, similar to what Aguilar-Bultet et al. [6] reported, ST14 and ST399 from CC14 clustered together, but ST91 (cluster formed by a group of 5 isolated from MN infections) was not grouped with the other CC14 strains (ST14/ST399 included 1 SI and 5 CNS isolates). These cases may be due to the limited ability of MLST to establish phylogenetic relationships. MLST uses fragments from seven core genes unlike cgMLST and the phylogenetic tree shown here, which is based on core genome SNPs; hence discrepancy among the classification methods may vary when whole genome sequences are used for phylogenetic relationship determination.

The distribution of 61 virulence-associated genes was also surveyed. Genes encoded in LIPI-1 were present in most of the isolates, with shorter sequences of *actA* in 45.7% of the isolates, mostly belonging to lineage 1 and associated with CNS cases. ActA is a transmembrane protein that contains two sets of proline-rich- repeats (PRR) and directs three separate events known: (1) actin polymerization independent of repeat regions; (2) initiation of movement dependent on the repeat regions and the amount of ActA; and (3) movement rate also dependent on the PRR [50]. Deletion of part of the sequence of *actA* gene is a feature that has been related to attenuated phenotypes in vitro, as *actA* deletion strains tested in pregnant mice and guinea pigs cause fetal infection with a significant delay, needing a bacterial load two log units higher than the wild type virulent strain [3,51,52]. Interestingly, here we found that some strains from, i.e., CC1, CC4, and CC6 (lineage 1) described as hypervirulent clones, harbor *actA* mutations that have been associated with decreased ability to spread cell- to- cell causing a reduction in the number of bacterial cells [3].

Likewise, LIPI-3 and LIPI-4 genes were also screened and were not found in isolates from lineage 2, 3, and 4 as reported previously [2,12,14], with an exception in an MN-associated isolate belonging to lineage 3, where five of six LIPI-3 genes were present. Similarly, LIPI-4 genes associated with CC4, which confers selective tropism for the CNS and fetal-placental organs [2], were found in 14 CCs from lineage 1 including CC1, CC2, CC87, CC217, CC382, and CC639, as well as in lineage 3 and 4 strains. This gene cluster was reported initially as exclusive of CC4, however recent studies have reported this pathogenicity island in strains belonging to other lineage 1 CCs [53]. The role of several genetic elements has been investigated to elucidate the relation of hypervirulence with the increased frequency of certain CC/ST that cause invasive listeriosis, however, it must also consider the nature of the host since delayed or hypovirulent clones still can affect patients with highly immunosuppressive comorbidities, as it has been demonstrated before [2,54].

CC14 and CC7, which were of special interest as they were found as hypervirulent and hypovirulent, respectively, in *G. mellonella* larvae [55] showed differences in virulence gene content when compared. Regarding pathogenicity islands, CC14 harbor only LIPI-1, while CC7 strains possessed LIPI-1 and SSI-1. Likewise, *vip*, an invasion-associated gene, was absent in CC7 but present in CC14. *inlF* and *inlJ* were present in all the CC7 strains, while only present in strains belonging to ST91 from CC14. Additionally, differences within CC14 sequence types were also apparent, not only when evaluating *inlF* and *inlJ* but also in *inlH* exclusively present in ST14 and ST399 (Figure 2 and Figure 3). These differences in gene content among STs from the same CC may contribute to explaining why ST91 and ST14/ST399 did not cluster together when the core SNP-based phylogeny tree was created (Figure 1).

Prior studies have shown a high degree of gene consistency within genotypes and have identified virulence genes and gene clusters associated with specific CC/ST [1,2,5]. Although we used draft genomes for our analyses, we consider that there is a very low probability that the absence of an evaluated virulence gene was due to an incomplete sequence. In our analyses, a gene was considered absent if: (1) the identity percentage between the query and the target sequences was <90%; (2) the gene was not found in the target sequence; (3) the gene was not complete (the maximum difference between the query and the target sequences was established as 170 nucleotides). The majority of our dataset includes different genomes that belong to the same CC/ST as we showed in Figure 2 and Figure 3, where for example there are 47 CC1 strains, and only 1.9% (4/47), have a slightly different virulence gene profile.

Although, *L. monocytogenes* virulence-associated genes present variations at the genetic level within the species, it is still considered highly clonal sharing around 70% of the genes among strains. The increased number of accessory genes (around 80% of the pan genome) is responsible for most of the strain-specific features even when variation in core virulence genes is present. Several studies have shown that isolates belonging to the same genotypic subgroup (CC/ST) often share the same virulence genes no matter the source of isolation [1,6,56] as shown in Figure 2 and Figure 3. Furthermore, while there is a differential distribution of CC/STs in human-associated and ruminant-associated isolates, major CCs such as CC1 and CC4 have been shown to spread globally causing most of the listeriosis cases in both hosts [2,11]. Thus, when several strains with highly conserved genomes are treated as independent, the variants that separate the subgroups may seem to be associated with a phenotype even with no causal link, due to the clonal nature of the bacterial populations [57]. To overcome this issue when conducting the bacterial GWAS in our study, we included strains from the same lineage/CC/ST with different phenotypes and from different hosts and geographic locations.

Using a gene-based GWAS, we identified orthologous genes of phage phiX174 associated with MN infections. Prophage genes have been found to confer increased virulence in *L. monocytogenes* strains in murine models, and to also play an important role in niche adaptation. There are three main bacteriophages that infect *L. monocytogenes*: A006, A500, and P35. These phages have genomes between 35.8 and 134.5 kb in size, with G + C contents between 35.5 and 40.8%. They belong to the Siphoviridae family and feature a similar genome organization, where open reading frames (ORFs) are organized into functional clusters that reflect the direction of transcription. Furthermore, integration sites in the bacterial genome revealed that both A500 and A006 specifically target the 3′ ends of tRNA genes [58].

Analysis of genomes from ST204 or ST121, for example, has shown that the majority of variations are linked to mobile elements such as plasmids, transposons, and phage insertions and that these elements were conserved in the ST population, suggesting that they may provide advantages in the diversity of niches where these STs are found [59,60]. Additionally, prophage regions were located adjacent to tRNAs, indicating that tRNAs are anchoring elements for the uptake of prophage DNA [61]. Diverse bacterial communities in the ‘farm to fork’ environments can also influence genetic diversity and contribute to the fact that genetic elements such as prophages could be horizontally transferred, conferring new functions like resistance to phagocytosis by macrophages, drug/sanitizer resistance mechanisms, increased biofilm formation, or adhesion to human cells [62,63]. We also identified genes coding the type I RM system, a group of DNA methyltransferases (MTases) that protect bacterial cells against phage infection while reducing horizontal gene transfer, thus it plays an important role in the ecology and evolution of bacteria [64]. This system can target foreign invading DNA with restriction endonucleases, and associated methyltransferases protect host DNA from restriction. It is also involved in the regulation of gene expression, helping bacteria to cope with environmental changes in nutrient availability, pH, temperature, and osmolarity [65].

Additionally, core-genome SNPs associated with CNS infections and SI were identified in an SNP-based GWAS. Interestingly, the SNPs found as associated with the two clinical outcomes are linked to environmental adaptation and virulence of *L. monocytogenes*. One of the SNPs associated with CNS cases (77,529T > C) was found in the gene that codes the phosphoenolpyruvate (PEP) mutase. This enzyme belongs to the isomerases family, specifically the phosphotransferases, whose main function is to transfer phosphate groups and form carbon-phosphorus bonds. Many enzymes that use pyruvate as a substrate are modulated by the phosphotransferase system (PTS) pathway used by bacteria for sugar uptake. Studies have shown that the lack of sigma 54 factor (*rpoN*), a subunit of bacterial RNA polymerase involved in nitrogen and carbon utilization, flagellar synthesis, and virulence directly modifies the PTS pathway affecting the pyruvate to PEP ratio, which influences the expression of pyruvate metabolism-related enzymes [66]. Furthermore, a mutation in one of the genes codifying the PTS sugar transporter subunit IIC (38,820C > A) was found associated with SI. This system transports carbon sources like glucose and cellobiose, specifically when the bacterium is outside the host cell, which may serve as an environmental signal to switch between an extracellular saprophyte to an intracellular pathogen [67]. The diversity of carbon sources in both hosts and environment, and the interaction with other microbes, may be an important factor for *L. monocytogenes* to maintain a diverse repertoire of sugar transporters to cope with these changes.

Likewise, substitutions in *rpoB* that encode the DNA-dependent RNA polymerase subunit beta (271,402A > G) and in a region of the *ccmA* gene (298,930C > T/A) that encoded an ABC- type multidrug transport system/ATPase component were identified. Mutations in these genes have been linked to rifampicin and rifabutin resistance in strains isolated from food products [68], and have been found to be important for bacterial adhesion, biofilm, and lipopolysaccharides biosynthesis [65]. Overall, treeWAS was able to identify genes and SNPs in significant association with the three clinical outcomes evaluated here.

## 5. Conclusions

In this study, WGS from listeriosis cases were evaluated in order to identify genetic determinants possibly involved in causing a particular clinical outcome (CNS infections, MN infections, and SI) in humans and ruminants. Our gene and SNP-based GWAS revealed 14 genes and five SNPs significantly overrepresented in one or more of the clinical outcomes evaluated. Those genetic variants play a key role in transcription, environmental adaptation, and virulence. Similar approaches have been already applied to find associations between genetic variants and antimicrobial resistance [69], growth in cold conditions [20], and host adaptation [5]. As such, an approach to screen for genetic markers, associated with a phenotype of interest, provides us with a narrow set of elements that could be evaluated further. Subsequent analyses are required to confirm that a causal relationship is truly present and to verify the role of the associated genes with the pathogenesis process of each clinical outcome. Moreover, the detection of isolates harboring phenotype-specific genetic markers could potentially reinforce preventive and control measures.

## Figures and Tables

**Table 1:** Genes associated with the three clinical outcomes evaluated by gene-based GWAS.

Locus ID	Gene Product	Gene Length (ncl)	Clinical Outcome	Number of Strains Found (n = 232) ^a^
EAL09991 *	tRNA-Phe(gaa)	74	CNS	195
WP_044683321	hypothetical protein/ phage capsid protein (phiX174)	117	MN	45
ENH11862	hypothetical protein/minor spike protein H (phiX174)	987	MN	45
NP_040712	hypothetical protein/major spike protein G (phiX174)	528	MN	45
ABN49622	hypothetical protein/replication initiation protein gpA (phiX174)	1406	MN	41
WP_016670801	hypothetical protein/phage protein C (phiX174)	144	MN	46
WP_000084700	hypothetical protein/phage protein D (phiX174)	459	MN	45
WP_000033471	hypothetical protein/DNA binding protein J (phiX174)	117	MN	49
EAL09991 *	tRNA-Phe(gaa)	73	MN	195
EAL09991	tRNA-Tyr(gta)	83	MN	209
BAO93225	tRNA-Ala(tgc)	75	MN	59
WP_003734550	type I restriction-modification system subunit M	2576	SI	30
WP_003743526	type I restriction endonuclease subunit R	3107	SI	30
WP_021496534	hypothetical protein	338	SI	30

* Gene shared by CNS and MN; ^a^ Gene was present in the strain independent of the clinical outcome classification

**Table 2:** SNPs associated with the three clinical outcomes evaluated by SNP-based GWAS.

Associated Position	Reference Nucleotide	SNP	Locus Tag	Gene Product	Protein ID	Region Start	Region End	Clinical Outcome
77,529	T	C	LMRG_RS00365	Phosphoenolpyruvate mutase	WP_014600361.1	76,760	77,533	CNS
271,402	A	G	LMRG_RS01320	DNA-directed RNA polymerase subunit beta	WP_003723046.1	268,638	272,243	CNS
38,820	C	A	LMRG_RS00170	PTS sugar transporter subunit IIC	WP_003721657.1	38,033	39,385	SI
271,402	A	G	LMRG_RS01320	DNA-directed RNA polymerase subunit beta	WP_003723046.1	268,638	272,243	SI
298,930	C	T/A	LMRG_RS01435	ABC transporter ATP-binding protein	WP_014600437.1	298,775	299,440	SI

## Data Availability

All sequence data is available in the Sequence Read Archive at NCBI. SRR accession numbers for each strain are provided in Table S1.

## Supplementary Materials

The following supporting information can be downloaded at: https://www.mdpi.com/article/10.3390/microorganisms10101934/s1, Figure S1: Manhattan plots from the gene-based GWAS using treeWAS; Figure S2: Manhattan plots from the SNP-based GWAS using treeWAS; Table S1: L. monocytogenes genomes from listeriosis cases associated with three main clinical outcomes; Table S2: Set of virulence-associated genes evaluated in this study.
