# Supplementary material for: Genome-Wide Association Study of Listeria monocytogenes Isolates Causing Three Different Clinical Outcomes
Source: Microorganisms. 2022 Sep 29;10(10):1934. doi: 10.3390/microorganisms10101934 (PMC9610272; doi:10.3390/microorganisms10101934)
Supplement: Supplementary file 1 [file microorganisms-10-01934-s001.zip › Figures S1,S2.pdf]

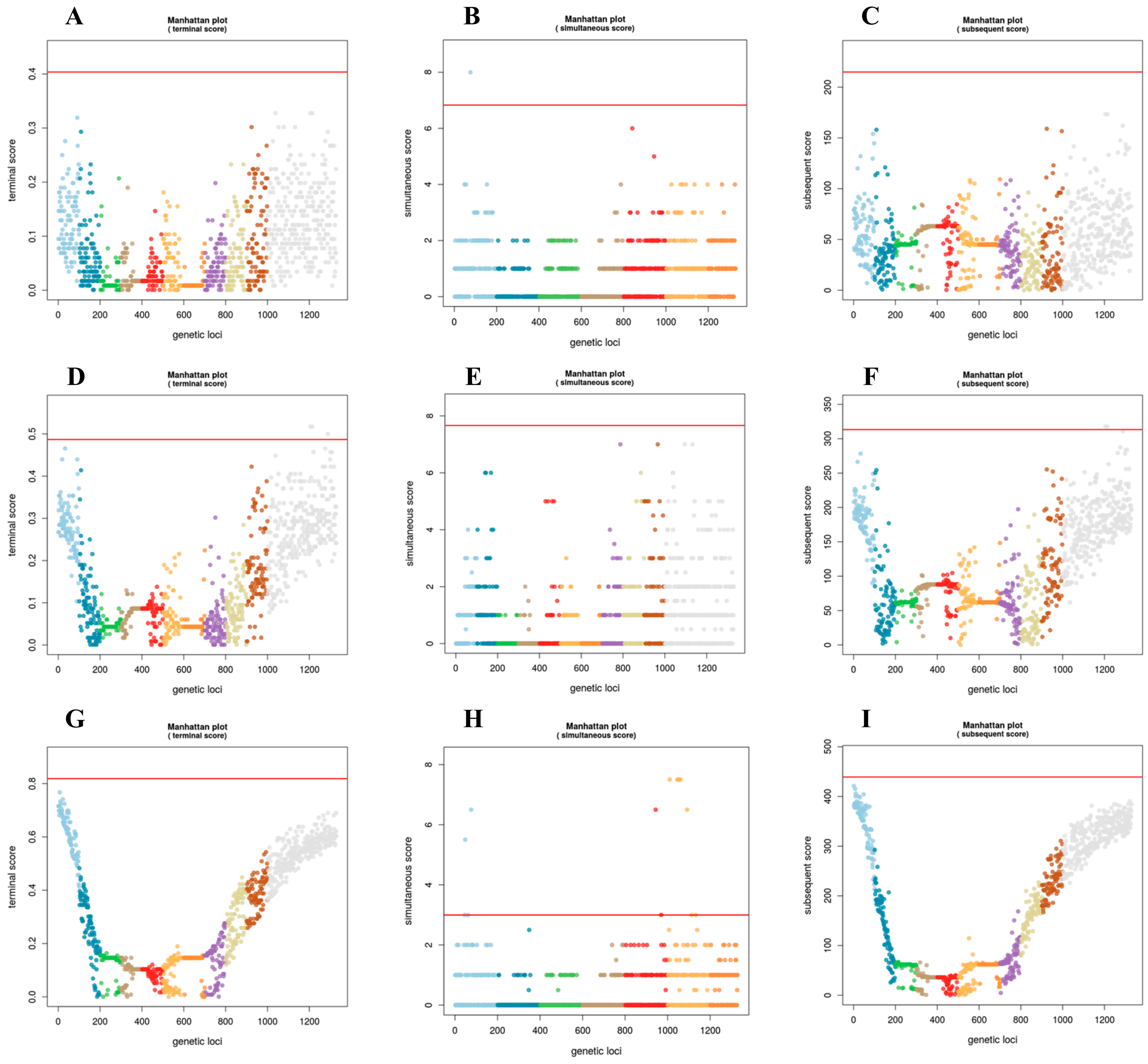

Figure S1. Manhattan plots from the gene-based GWAS using treeWAS. (A, D, G) Score 1, (B, E, H) Score 2, and (C, F, I) Score 3 showing association score values for CNS (A, B, C), SI (D, E, F), and MN (G, H, I) and accessory genes. Dots above significant threshold (red line) indicate significant associations.

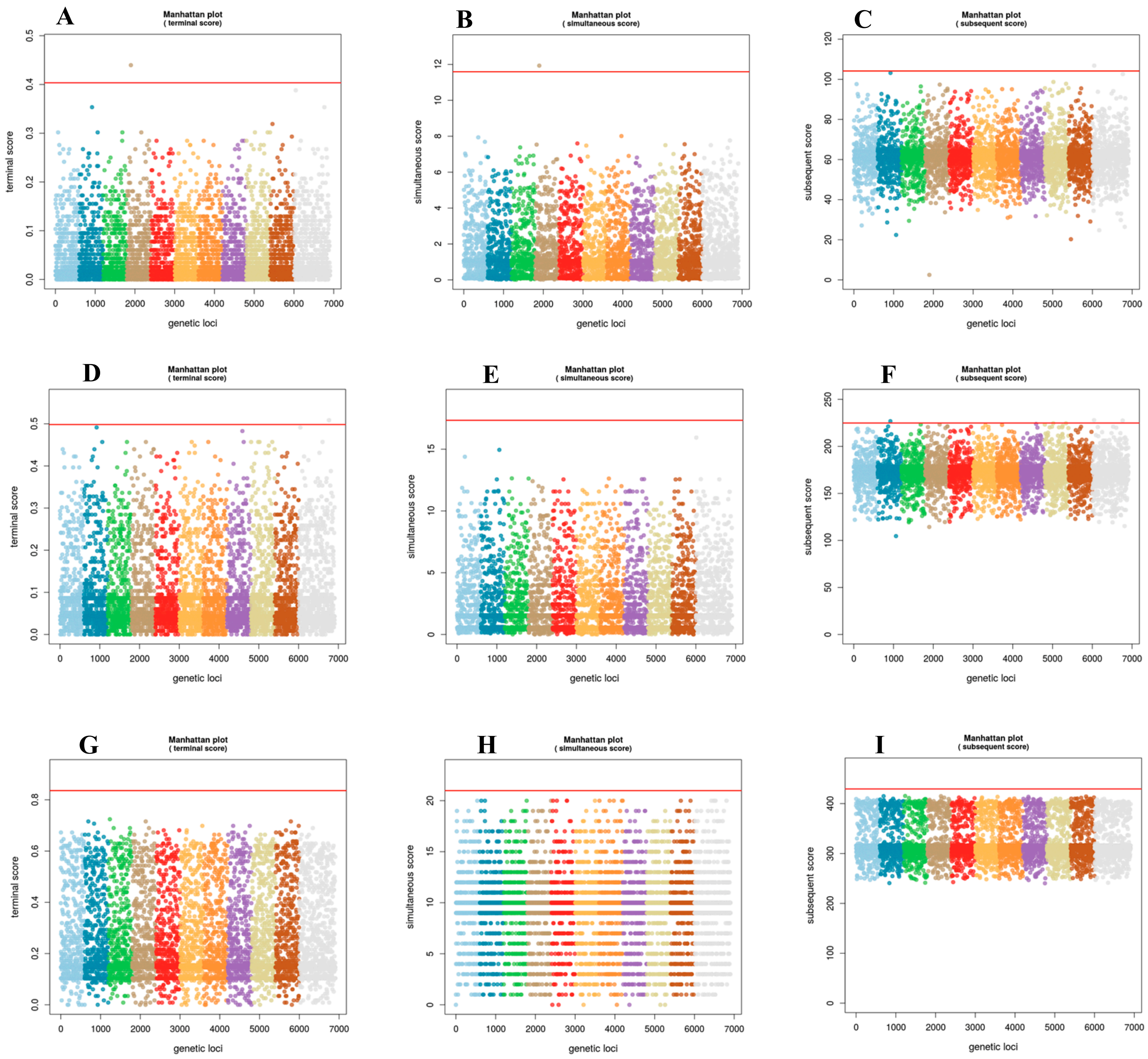

Figure S2. Manhattan plots from the SNP-based GWAS using treeWAS. (A, D, G) Score 1, (B, E, H) Score 2, and (C, F, I) Score 3 showing association score values for CNS (A, B, C), SI (D, E, F), and MN (G, H, I) and all core-SNPs. Dots above significant threshold (red line) indicate significant associations.
