# Supplementary material for: Genome-Wide Association Study of Listeria monocytogenes Isolates Causing Three Different Clinical Outcomes
Source: Microorganisms. 2022 Sep 29;10(10):1934. doi: 10.3390/microorganisms10101934 (PMC9610272; doi:10.3390/microorganisms10101934)
Supplement: Supplementary file 1 [file microorganisms-10-01934-s001.zip › Table S2.pdf]

Table S2. Virulence genes screened in the *L. monocytogenes* dataset

Gene	Symbol/Aliases	Gene product	Accession number/Location	Pathogenicity Island
actA	lmo0204	actin-assembly inducing protein precursor	NC_003210.1:209470-211389	LIPI-1
hly	lmo0202	listeriolysin O precursor	NC_003210.1:205819-207408	
mpl	lmo0203	Zinc metalloproteinase precursor	NC_003210.1:207739-209271	
inlA	lmo0433	internalin A	NC_003210.1:454534-456936	
inlB	lmo0434	internalin B	NC_003210.1:457021-458913	
plcA	lmo0201	phosphatidylinositol-specific phospholipase c	NC_003210.1:c205577-204624	
plcB	lmo0205	phospholipase C	NC_003210.1:211425-212294	
prfA	lmo0200	listeriolysin positive regulatory protein	NC_003210.1:c204353-203640	
LMOf2365_1113	LMOf2365_1113	ABC transporter, ATP-binding protein	NC_002973.6:1126911-1127798	LIPI-3
LMOf2365_1114	LMOf2365_1114	ABC transporter permease	NC_002973.6:1127798-1128547	
LMOf2365_1115	LMOf2365_1115	hypothetical protein	NC_002973.6:1128588-1128905	
LMOf2365_1116	LMOf2365_1116	SagB/ThcOx family dehydrogenase	NC_002973.6:1128902-1129777	

Table S2. Virulence genes screened in the *L. monocytogenes* dataset (continued)

Gene	Symbol/Aliases	Gene product	Accession number/Location	Pathogenicity Island
LMOf2365_1117	LMOf2365_1117	hypothetical protein	NC_002973.6:1129785-1130702	
LMOf2365_1118	LMOf2365_1118	streptolysin associated protein SagD	NC_002973.6:1130695-1132008	
Lm4b-02327	LM4B_RS11760	PTS lactose/cellobiose transporter subunit IIA	NC_012488.1:2396192-2396506	LIPI-4
Lm4b-02328	LM4B_RS11765	PTS sugar transporter subunit IIB	NC_012488.1:2396506-2396862	
Lm4b-02329	LM4B_RS11770	PTS sugar transporter subunit IIC	NC_012488.1:2396882-2398183	
lmo0444	lmo0444	hypothetical protein	NC_003210.1:473936-476716	
lmo0445	lmo0445	122ranscriptional regulator	NC_003210.1:476960-478447	
lmo0446	lmo0446	penicillin acylase	NC_003210.1:478721-479710	SSI-1
lmo0447	lmo0447	glutamate decarboxylase	NC_003210.1:479765-481153	
lmo0448	lmo0448	amino acid antiporter	NC_003210.1:481250-482701	
ami	lmo2558	autolysin, amidase	NC_003210.1:2635167-2637920	
aut	lmo1076	autolysin	NC_003210.1:1106041-1107759	

Table S2. Virulence genes screened in the *L. monocytogenes* dataset (continued)

Gene	Symbol/Aliases	Gene product	Accession number/Location	Pathogenicity Island
ctaP	lmo0135	peptide ABC transporter substrate-binding protein	NC_003210.1:137323-138897	
fbpA	lmo1829	fibronectin-binding proteins	NC_003210.1:1904152-1905864	
flaA	lmo0690	flagellin	NC_003210.1:724896-725759	
fri	lmo0943	non-heme iron-binding ferritin	NC_003210.1:979059-979529	
gap	lmo2459	glyceraldehyde-3-phosphate dehydrogenase	NC_003210.1:c2532320-2531310	
gtcA	lmo2549	wall teichoic acid glycosylation protein GtcA	NC_003210.1:c2625417-2624980	
hfq	lmo1295	host factor-1 protein	NC_003210.1:1323450-132368	
inlC	lmo1786	internalin C	AL591981.1:107152-108104	
inlF	lmo0409	Internalin F	NC_003210.1:429630-432095	
inlH	lmo0263	internalin H	NC_003210.1:284365-286011	
inlJ	lmo2821	internalin J	NC_003210.1:2907153-2909708	
inlP	lmo2470	Internalin P	NC_003210.1:2544267-2545433	

Table S2. Virulence genes screened in the *L. monocytogenes* dataset (continued)

Gene	Symbol/Aliases	Gene product	Accession number/Location	Pathogenicity Island
lap	lmo1634	bifunctional acetaldehyde-CoA/alcohol	NC_003210.1:1677409-1680009	
lapB	lmo1666	peptidoglycan-linked protein	NC_003210.1:1717193-1722328	
lgt	lmo2482	prolipoprotein diacylglyceryl transferase	NC_003210.1:c2558044-2557211	
lntA	lmo0438	hypothetical protein (LntA)	NC_003210.1:467519-468136	
lpeA	lmo1847	metal ABC transporter	NC_003210.1:1923825-1924757	
lplA1	lmo0931	lipoate protein ligase A	NC_003210.1:967784-968779	
lspA	lmo1844	lipoprotein signal peptidase	NC_003210.1:1920436-1920900	
mprF	lmo1695	hypothetical protein (MprF)	NC_003210.1:1758694-1761291	
murA	lmo2691	autolysin	NC_003210.1:2766935-2768707	
oppA	lmo2196	peptide ABC transporter substrate-binding protein	NC_003210.1:2284539-2286215	
pgdA	lmo0415	endo-1,4-beta-xylanase	NC_003210.1:437482-438882	
pgl	lmo0558	hypothetical protein (Pgl)	NC_003210.1:596580-597620	

Table S2. Virulence genes screened in the *L. monocytogenes* dataset (continued)

Gene	Symbol/Aliases	Gene product	Accession number/Location	Pathogenicity Island
prsA2	lmo2219	foldase	NC_003210.1:2306833-2307714	
pycA	lmo1072	pyruvate carboxylase	NC_003210.1:1099266-1102706	
recA	lmo1398	recombinase A	NC_003210.1:1425419-1426465	
relA	lmo1523	(p)ppGpp synthetase	NC_003210.1:c1558364-1556148	
secA	lmo2510	preprotein translocase subunit SecA	NC_003210.1:c2589813-2587300	
sigB	lmo0895	RNA polymerase sigma factor SigB	NC_003210.1:930671-931450	
sipZ	lmo1271	type I signal peptidase	NC_003210.1:1294360-1294902	
sod	lmo1439	superoxide dismutase	NC_003210.1:c1473588-1472980	
srtA	lmo0929	sortase	NC_003210.1:966245-966913	
svpA	lmo2185	hypothetical protein (SipZ)	NC_003210.1:2272403-2274112	
uhpT	lmo0838	sugar:phosphate antiporter	NC_003210.1:869095-	
vip	lmo0320	peptidoglycan-bound surface protein	NC_003210.1:344850-346049	

Table S2. Virulence genes screened in the *L. monocytogenes* dataset (continued)

Gene	Symbol/Aliases	Gene product	Accession number/Location	Pathogenicity Island
virR	lmo1745	two-component response regulator	NC_003210.1:1814403-1815080	
